# Service providers’ perspectives on the challenges of informal caregiving and the need for caregiver-orientated mental health services in rural South Africa: A descriptive study

**DOI:** 10.1371/journal.pone.0309090

**Published:** 2024-08-23

**Authors:** Olindah Silaule, Nokuthula Gloria Nkosi, Fasloen Adams

**Affiliations:** 1 Occupational Therapy Department, University of the Witwatersrand, Johannesburg, South Africa; 2 Division of Occupational Therapy, University of Cape Town, Cape Town, South Africa; 3 Department of Nursing Education, University of the Witwatersrand, Johannesburg, South Africa; 4 Occupational Therapy Department, Stellenbosch University, Stellenbosch, South Africa; Ensign Global College, GHANA

## Abstract

Informal caregivers of persons with mental disorders encounter various challenges in their role of caregiving. As such, they require support to enable them to cope with the demands of their caregiving. There is comprehensive evidence on the experiences of burden among informal caregivers in mental health; however, there is a limited number of studies that report on the mental health services aimed specifically at supporting informal caregivers in their role. To address this gap, this study aimed to explore the perspectives of the service providers regarding the challenges encountered by informal caregivers and the mental health services available to support these caregivers. Semi-structured interviews were conducted with mental health coordinators at provincial, district, and sub-district level and mental health professionals from a district hospital. Focus group discussions were conducted with primary healthcare supervisors and community health workers in Bushbuckridge municipality, South Africa at participants’ workplaces and sub-district offices. Semi-structured interviews and focus group guides with semi-structured questions were used to direct data collection in August 2022–January 2023. All interviews were audio recorded and transcribed verbatim. Reflexive thematic inductive analysis was conducted using NVivo 12 software. Three themes were identified, namely perceived caregiving consequences and related factors, current state of mental health services, and factors affecting delivery of informal caregiver mental health services. The service providers acknowledged the negative consequences faced by informal caregivers. This includes the experience of caregiver burden which was attributed to the uncooperative and violent behaviours exhibited by the mental health care users. The current state of formal and informal community mental health services was described and considered inadequate to meet informal caregivers’ needs. Various personal, health system, and contextual factors influencing the provision of caregiver-orientated services were identified. The findings revealed the need for intersectoral collaborations between hospital-based and community-based mental health service providers, and community stakeholders to ensure provision of user-friendly and accessible mental health services for informal caregivers.

## Introduction

Globally, mental disorders are highly prevalent and ranked as the fifth highest contributor to overall disease burden [1]. It is estimated that more than 80% of people with mental disorders reside in low- and middle-income countries (LMICs) [2]. Severe mental disorders (SMDs), including depressive disorders, schizophrenia, bipolar mood disorders, and substance use disorders, are reported to be among the top 10 causes of disability in LMICs, accounting for 19.1% of all disabilities and health related conditions [2]. The growing burden of mental disorders is a continuous challenge in LMICs because mental health remains a neglected priority, especially in rural areas [1, 3]. A mental health treatment gap of 75–85% persists between the need and provision of treatment in LMICs compared to the 30–50% gap in high income countries [1, 4]. It is estimated that only 10.4% of persons with SMDs in Nigeria and 11.1% in China receive treatment [5]. In South Africa, only 27% of persons with SMDs are reported to receive treatment, which is concerning as 52% of the South African population resides in rural areas, and it can be assumed that most persons with SMDs who live in rural communities have limited mental health resources [6, 7]. Various factors have been identified to influence the provision of mental health services in LMICs like South Africa, such as infrastructural problems, shortage of mental health professionals, limited available health budget, affordability and accessibility of mental health services, public and personal stigma, and mental healthcare users’ (MHCUs) low perceived need for treatment [3, 8]. In LMICs, help-seeking and access to mental health services are further influenced by cultural and religious factors because many people are likely to seek help from complementary mental health service providers such as traditional, faith, and spiritual healers [9]. It is for this reason that calls have been made to integrate collaborative care in mental healthcare, particularly in resource-constrained contexts, and to ensure user-friendly services to improve outcomes for persons with mental disorders [10, 11].

In resource-constrained countries like South Africa, it is recommended that efforts be directed towards strengthening community-based mental health services to increase access [12]. Community mental health services are divided into formal and informal mental healthcare. Formal community mental health services are provided by primary healthcare workers employed by the department of health and social development [13], including care in day centres, rehabilitation facilities, community centres, home help, and outpatient services at primary healthcare facilities [13]. Informal community mental health services are non-specialised services provided by other sectors other than health and/or social development services, including care from families, friends, traditional healers, religious organisations, village workers, lay community workers, and non-governmental organisations [13, 14]. Informal community mental health services are deemed essential in the management of SMDs as they provide cost-effective mental healthcare, reduce relapses for patients, and increase support for caregivers [15].

Despite continuous advocacy to strengthen community mental health services in LMICs, these services remain underdeveloped [16]. In South Africa, community mental health services remain under-funded and under-resourced as the limited mental health funds are directed towards hospital-based care [17, 18]. It should be acknowledged that the mental health system, provided by the Department of Health in South Africa, fails to meet the diverse needs of patients with mental disorders [7]. These unmet needs have a spill-over effect on informal caregivers, including families, friends, and community members who provide care to MHCUs without remuneration and with limited support in their role [19]. The National Mental Health Policy framework [20] recognises the support that MHCUs and their family or caregivers requires, but not much has been achieved in this regard [18]. Given the current state of mental health services in South Africa, informal caregivers are a vital resource in the care and management of persons with mental disorders [21]. Informal caregivers are expected to deal with patient behaviour, structure their routines, and monitor treatment without any access to resources for support [22–24]. For example, Vigo et al. [25] highlight lack of psychoeducation, communication, involvement in treatment planning, and community social services as precipitating factors of caregiver burden.

Previous studies conducted in rural South Africa reveal high levels of burden among informal caregivers of persons with SMDs [26–29]. Identified burdens include emotional and physical burdens, economic burdens, disrupted family routines, and relationship burdens [29, 30]. Therefore, informal caregivers must be supported to ensure the care and recovery of patients within communities [31]. In addition, Adelman et al. [32] suggest that services aimed at caregivers should incorporate psychoeducation, communication, and emotional support. While previous studies reported on the challenges of caregiving in mental health, very limited studies report on these challenges from the service providers’ perspectives. In addition, there is limited studies reporting on the mental health services aimed at supporting informal caregivers in their role. Understanding the perspectives of the service providers regarding the challenges of informal caregiving is fundamental for the development and implementation of caregiver-orientated mental health services. It is for this reason that this study focused on exploring the perspectives of the service providers regarding the challenges of informal caregiving and the mental health services for supporting the informal caregivers in rural South Africa.

## Materials and methods

A qualitative descriptive research design was adopted in this study. Semi-structured interviews were conducted with mental health coordinators at provincial, district, and sub-district level and mental health professionals from a district hospital in Bushbuckridge municipality in South Africa [33]. Focus group discussions were also conducted with primary healthcare supervisors and community health workers (CHWs) based in the local communities of Bushbuckridge municipality [33]. Data were collected sequentially according to the various levels of healthcare where the participants worked, namely the province, district, and sub-district level. This article adhered to the Consolidated Criteria for Reporting Qualitative Research (COREQ) [34].

### Setting

This study took place in Bushbuckridge municipality situated in Ehlanzeni district in the north-eastern rural part of Mpumalanga province, South Africa. This municipality has a population size of 546 215 [35]. There is one regional hospital, two district hospitals, and 34 primary health clinics situated within Bushbuckridge municipality. Currently, the regional and one district hospital offer 72-hour observation services, and the other district hospital has a mental health unit, which is the largest in the entire Mpumalanga province with a 50-bed capacity. This mental health unit offers in- and outpatient services to MHCUs with variety of mental disorders, ranging from acute to chronic and forensic cases. Therefore, all persons with SMDs requiring long-term and/or in-patient care are referred to this facility for further management. Once discharged to the care of their families, MHCUs are referred to primary health clinics to collect their psychotropic medications. The primary health clinics are organised into clusters with five clinics per area. A cluster refers to a group of clinics supervised by the same primary healthcare supervisor [36]. The clinics are nursed-led.

### Participants

Twenty-nine mental health service providers were purposively selected to participate in this study. To be selected for this study, participants needed to be over 18 years of age and be employed in their current position for longer than six months.

### Data collection

Once all relevant consent and permissions were obtained, data were gathered between August 2022 and January 2023 using semi-structured interviews and focus groups. Conducting individual semi-structured interviews and focus groups was necessary for capturing the perspectives of multiple service providers across the various levels of care. A focus group was considered a convenient and cost-effective way of gathering data from the service providers. This allowed for an in-depth exploration of the challenges experienced by informal caregivers at the community level and the current mental health services for supporting informal caregivers.

The first author (OS), who served as the primary investigator conducted semi-structured interviews under the guidance of two research supervisors (FA and NGN). Prior to data collection, the PI attended a qualitative methodology workshop, which equipped her with skills to conduct semi-structured interviews and qualitative data analysis using an NVivo software. Upon the commencement of data collection, the primary investigator contacted all potential participants telephonically, informed them of the purpose of the study, and invited them to participate. All approached participants agreed to engage in the study. A convenient date, time, and place were identified for the interviews; the semi-structured interviews ran for a maximum of one hour. All participants preferred that the interviews be conducted in their workplace, which allowed for flexibility and an in-depth understanding of the context. Interviews were conducted in a private space to give the participants an opportunity to freely express themselves.

Prior to the interviews, the researcher went through the information sheet detailing the purpose of the study. Three semi-structured interviews were conducted with four mental health coordinators at the provincial, district and sub-district level. This was followed by four semi-structured interviews that were conducted with the mental health professionals including a medical officer, nursing manager, a social worker and two occupational therapists. Of the seven interviews conducted, one was conducted with two mental health coordinators, and another was conducted with two occupational therapists. These interviews were conducted together for the convenience of the participants. Data were collected until saturation was reached. To assess data saturation, code frequency counts were employed by reviewing each interview transcript following each interview and keeping track of the new codes in each transcript to determine their frequency. Data saturation was assumed once the frequency of the new codes diminished [37]. An interview guide and demographic questionnaire were used. The development of the demographic questionnaire and interview guide was informed by literature [38]. Participants were invited to share their views on the challenges of informal caregiving and the mental health services for supporting the informal caregivers in their role. The first open-ended question aimed to capture the generic perspectives of the service providers on the observations they have made about the people who are taking care of people with mental disorders within the community. This was followed by questions aimed specifically at exploring the service providers’ perspectives regarding the burden of care and the formal and informal community mental health services for supporting informal caregivers of persons with SMDs.

For convenience to the participants, two focus group discussions were conducted concurrently, thus requiring two separate teams to facilitate. The first team comprised of the primary investigator and the minute taker, and the second team had a skilled focus group facilitator and a minute taker. The primary investigator arranged for the focus groups through the Bushbuckridge sub-district coordinator. The focus groups were planned to coincide with the primary healthcare supervisors’ monthly meeting. Two focus groups were conducted with the primary healthcare supervisors and CHWs. The primary investigator facilitated a focus group with the CHWs, and an identified skilled moderator facilitated the focus group with the primary healthcare supervisors. To ensure uniformity, the two focus group facilitators had meetings prior to data collection to discuss the processes that were to be to be followed during the discussions. A semi-structured focus group guide was developed to ensure consistency in the facilitation of the focus groups and to guide the exploration of the perspectives of the participants regarding the challenges of informal caregiving and the mental health services for supporting informal caregivers. To ensure validity of the questions the focus group guide was reviewed by four experts, which includes two lecturers, a staff nurse and an occupational therapist who are experienced in mental health. The discussions started with an open-ended question aimed to capture perspectives of the participants on the observations they have made about the people who are taking care of people with mental disorders within the community. This was followed by questions aimed specifically at exploring the service providers’ perspectives regarding the burden of care and the formal and informal community mental health services for supporting informal caregivers of persons with SMDs. The sessions ran concurrently in separate venues within the Bushbuckridge sub-district offices. Data collection ceased after 1.5 hours when data saturation was reached i.e. when there was limited new information emerging from the discussion in the group. Debriefing was done between the two teams who conducted the focus groups and field notes were captured to add to the details of the interviews. Prior to data collection, written informed consent was obtained from all participants to grant permission for participation and audio recording of the interviews and focus groups. The interviews and one focus group were conducted in English. The CHWs’ focus group was conducted in Xitsonga, which is the first language of the participants and interviewer. All interviews were audio-recorded, transcribed, and translated to English by a language specialist who works as a researcher at a research entity situated in Bushbuckridge municipality. In preparation for data analysis, the primary investigator listened to the audio recording while concurrently reading the transcripts. Where necessary corrections and omissions were addressed to ensure accuracy in data translation.

### Data analysis

Transcriptions were uploaded onto NVivo 12 software for analysis. A six-phase reflexive thematic inductive approach proposed by Braun and Clarke [39] was used to systematically analyse the data. This enabled the researchers to find meanings and patterns within the data [39]. The primary investigator (OS) conducted data familiarisation by reading and re-reading the transcripts while concurrently listening to the recordings. Familiarisation notes were made, and these provided guidance for generating ideas for codes. Systematic coding was conducted inductively by the PI, and each code was assigned a description to ensure consistency. The PI then grouped the generated codes together to form patterns that led to the generation of initial subthemes and themes. The refining and renaming of themes were done by reading the coded extracts to ensure they merged coherently into a pattern to form a specific theme. Additionally, the data set and familiarisation notes were read to ensure no codes were missed and to check the validity of the themes. To ensure trustworthiness, an audit trail of the analysis process was conducted by an experienced qualitative researcher. To promote the quality and truthfulness of findings, peer briefings and presentation of findings were done with the two research supervisors (FA and NGN). Member checking was conducted by sharing the transcripts with the participants who were then given an opportunity to comment and add any information that they feel may have been omitted. Keeping a reflexive journal enabled the primary investigator to capture all the field notes outlining the thoughts and observations that guided the analysis process.

### Ethical considerations

The Human Research Ethics Committee of the University of the Witwatersrand (M200957) approved this study. Permission was also sought from the Mpumalanga Department of Health Research Committee (MP_202010_010). The methods followed in this study were in accordance with the relevant ethical principles for medical research involving human subjects [40]. Prior to data collection, all participants were given an information sheet that informed them about the aim of the study, confidentiality, and anonymity. Informed written consent was obtained from all participants to grant permission to participate and audio record the interviews.

## Results

### Participant characteristics

The participant characteristics are presented in Table 1. Overall, 29 participants participated in this study, including four mental health coordinators at the provincial, district, and sub-district level; five mental health professionals at the mental health unit of a district hospital situated within Bushbuckridge municipality; five primary healthcare supervisors; and 15 CHWs. The participants’ years of experience ranged between 2 and 34 years.

**Table 1:** Participant characteristics.

Identity of Participant	Format of data collection	Setting of data collection	Number
**Mental health coordinators (MHCs)**			
Provincial	SSI	Workplace	2
District	SSI	Workplace	1
Sub-district	SSI	Online	1
**Mental health professionals (MHPs)**			
Medical officer	SSI	Workplace	1
Nursing manager	SSI	Workplace	1
Social worker	SSI	Workplace	1
Occupational therapist	SSI	Workplace	2
**Community-based service providers**			
Primary healthcare supervisors (PHCSs)	FG	Sub-district offices	5
CHWs	FG	Sub-district offices	15
**Total**			29

Abbreviations: SSI = semi-structured interviews; FG = focus groups

The study aimed to explore the perspectives of the service providers regarding the challenges of informal caregiving and mental health services available for supporting informal caregivers of persons with SMDs in rural South Africa. The analysis of the semi-structured interviews and focus groups led to identification of the following three themes, which are set out in Table 2:

- Theme 1: Perceived caregiving consequences and related factors, which outlines service providers’ perspectives on the demands encountered by informal caregivers and contributing factors.
- Theme 2: Current state of mental health services, which outlines the formal and informal mental health services currently offered at the study site and the suitability of these services to alleviate burden of care among informal caregivers.
- Theme 3: Factors affecting the delivery of caregiver mental health services, which outlines factors currently hindering the provision of mental health services for alleviating burden among informal caregivers at the study site.

**Table 2:** Themes and subthemes representing service providers’ perspectives on informal caregiving.

Themes	Subthemes
Perceive caregiving consequences and related factors	Consequences of caregiving
	Caregiver-related factors impacting caregiving
	Contextual factors impacting caregiving
	Health system factors impacting caregiving
	Intersectoral factors impacting caregiving
Current state of mental health services	Current community-based mental health services
	Current institution-based mental health services
Factors impacting delivery of caregiver mental health services	Caregiver-related factors impacting delivery of caregiver mental health services
	Mental health professionals’ factors impacting delivery of caregiver mental health services
	Contextual factors impacting delivery of caregiver mental health services

The voices of the participants are reflected in this paper by using direct quotes.

#### Theme 1: Perceived caregiving consequences and related factors

The service providers expressed that informal caregivers of persons with SMDs encounter challenges in their role. The informal caregivers are perceived to encounter negative caregiving consequences. Various factors were identified to affect the informal caregivers in their role including caregiver, contextual, health system, and intersectoral factors.

*Consequences of caregiving*. The service providers reported that informal caregivers encountered negative consequences in their caregiving. Identified negative consequences included caregiver burden. Identified burdens were attributed to the uncooperative and violent behaviours exhibited by the MHCUs. Some MHCUs were identified to abuse substances and often refused medications. Subsequently, MHCUs relapse and start displaying aggressive and violent behaviours towards their caregivers, making it difficult for them to provide care. This was captured in quotes like the following:

“The biggest challenge is smoking, and there are many substances that they smoke.” (MHP, SSI)

“I had a client who presented psychosis; the caregiver couldn’t take what was happening. They were being beaten by the relative with mental illness, and they struggled to bring him for consultation as he was not adhering to medication.” (Mental health coordinator, SSI)

Service providers expressed that some informal caregivers encountered stigma in the community, which leads to conflict. Community members require informal caregivers to account for the behaviour of the MHCUs and pay for damages caused by the MHCU. For example, one participant said: “Another challenge is that they have conflicts with the community because the mentally ill person may go out and destroy people’s things, and the community will come to the caregiver to report that their person has destroyed their things, and the caregiver will have to pay” (PHCS, FG). The stigma in the community means that some informal caregivers are likely to be lonely and isolated. A participant explained that “caregivers can feel very alone and even isolated within their families, and people don’t want to visit when there is someone with mental health issues in the house” (MHP, SSI).

Some MHCUs require full-time care, and as a result, the informal caregivers are restricted in terms of the activities they can do. Service providers indicated that some informal caregivers are forced to leave school or jobs to provide full-time care to a person with a mental disorder. The loss of income perpetuates the cycle of financial strain experienced by informal caregivers. For example, a participant said, “Her daughter is good with her [mother]. The problem is that she had to drop out of school because she wanted to take care of her mother” (CHW, FG).

The burden encountered by informal caregivers result in negative consequences that influence their emotional, mental, and physical well-being. The disruptive behaviours displayed by MHCUs cause informal caregivers to live in a constant state of fear for their safety, and consequently, they experience negative emotions such as frustration, anger, and stress due to their caregiving role. A service provider said: “They don’t feel free; they feel they are at high risk of getting killed by these patients. The burden is quite high on the caregivers, and many times you observe that the caregivers are very stressed all year around. They are frustrated and angry” (MHP, SSI).

The service providers perceived that the stress of caregiving compromises the mental health of informal caregivers. Some service providers reported that informal caregivers often present with mental conditions, including depression, anxiety and adjustment disorders, and one participant explained that “we are having these caregivers that are coming to consult mostly for depression and anxiety” (MHC, SSI). Some informal caregivers have also developed physical conditions due to the demands of caregiving. One service provider said, “The mother of the patient got sick and was diagnosed with high blood pressure and heart disease” (CHW, FG).

*Caregiver-related personal factors affecting caregiving*. According to the service providers, some informal caregivers lack the information and skills to handle the MHCUs, and as a result, they struggle to cope with the demands of caregiving. In addition, informal caregivers struggle to accept the conditions of MHCUs, which results in the exclusion and isolation of the MHCUs. One participant explained the problem as follows:

“The problem is lack of information; they don’t know how to take care of the mentally ill patient. They do not know how to handle them; they do not know how to talk to them; and that is the problem. They are afraid of them, and as long as they are mentally ill, they don’t treat them the same as others. They exclude them, they isolate them from all activities.” (MHC, SSI)

Lack of information leads some informal caregivers to delay seeking help for the MHCUs. The service providers reported that some caregivers sought help only when they are threatened by the MHCUs’ violent behaviour. This could be related to the fact that caregivers lack knowledge and understanding of the symptoms to look for that could indicate a decline or beginning of a psychotic episode, and thus, they only seek help when their lives are endangered. It was explained as follows: “You only see them when the patient is causing problems at home, or they call us and when we ask how we can assist them, they tell us that this guy [MHCU] is threatening us, breaking windows” (MHC, SSI).

The service providers also stated that to cope with the demands of caregiving, some informal caregivers adopt avoidant coping strategies. Due to the violent behaviours MHCUs displayed prior to hospitalisation, some caregivers avoid visiting them during hospitalisation as they fear the early discharge of the MHCUs. One participant said, “Caregivers are saying that they are violent and that is why they want them to be cared for in the hospital. They feel frustrated to take them home. Sometimes they end up not visiting the patient because they are scared that the healthcare workers will give them the patients to take them home” (MHP, SSI).

*Contextual factors impacting on caregiving*. The service providers identified the challenges encountered by informal caregivers to be related to the shortage of rehabilitative activities for MHCUs within the community. Service providers highlighted that hospitals play a key role in stabilising psychiatric conditions; however, there are very limited resources to rehabilitate MHCUs within their communities, and this poses a challenge as some require full-time care from their caregivers. For example, one participant said the following:

“There are no support groups in the communities, and if they were there, maybe they would have activities like soccer or any other games to be involved in … so there is nothing for them. We just give them the treatment, and they go back home until we see them again when they relapse, and this means that there is nothing for these patients when they are in the community.” (MHP, SSI)

The lack of support from family and the community were identified as a limitation in the context, affecting the informal caregivers. Although some communities hold community dialogues to discuss the care of people with mental disorders, none of these discussions are aimed specifically at creating support for the caregivers; for example, “the community dialogues are happening for the patients, but not to support the caregivers” (PHCS, FG).

*Health system factors impacting on caregiving*. The limited access to caregiver-orientated mental health services was identified as a factor that influences informal caregivers’ ability to cope with their caregiving. The service providers highlighted a general neglect of mental health services within the community. Despite acknowledging the importance of caregiver support, none of the community clinics facilitated mental health support groups aimed at supporting the informal caregivers: “We are not doing the support groups in our facilities, and this field [mental health] is usually neglected” (PHCS, FG).

Some participants expressed that services are targeted specifically at the MHCUs and that less focus is directed at meeting the caregivers’ needs. Lack of caregiver empowerment services was identified as a factor that compromises caregivers’ coping mechanisms. The service providers indicated that having patient-orientated services neglected the needs of the informal caregivers, and as a result, they lack information on handling MHCUs and are often perceived as less proactive and more passive because they accept the behaviours exhibited by the MHCU even at the detriment of their health. The following comments by participants highlight this:

“The services are only targeting the patient; the family don’t get these services.” (MHC, SSI)

“I think they become quite passive, and they just accept it as it is, and they are not proactive, and they are not empowered at all to change the circumstances.” (MHP, SSI)

*Intersectoral factors impacting on caregiving*. The service providers acknowledged that the provision of comprehensive mental health services requires collaboration between sectors such as health and social welfare, and as such, they identified some of the intersectoral factors they perceived as influencing informal caregivers. The service providers identified lack of long-term placements and day care centres as a factor that increases caregiving demands because it means that caregivers do not have time away from the MHCU. Caregivers are obligated to provide care even when their coping is compromised, and this increases their stress levels. One participant explained, “I think what is really hard is that we don’t have any options for places for the patients to go when caregivers are not coping anymore or a long-term placement, and that causes extra stress” (MHP, SSI).

Information regarding available support is not readily available for informal caregivers, and some informal caregivers complained about the lack of information on social grants and welfare support available to alleviate their financial strain. A participant explained their experience by saying, “I don’t think they are aware that they should also be getting the grants. Those who are aware, are the ones who are looking after the elderly people. They are getting the supplement grant or grant in aid. But, the caregivers of people with mental disorders, they don’t know about it” (PHCS, FG).

#### Theme 2: Current state of mental health services

Understanding the current state of mental health services was important to determine the level of support accessible to informal caregivers within their communities. Service providers described formal and informal community mental health services offered at the community and institution-based levels and their suitability to meet the needs of informal caregivers.

*Community-based mental health services*. Due to lack of physicians and psychiatrists, formal mental health services at primary healthcare clinics rely mostly on psychiatric nurses. The nurses are responsible for administrating psychotropic medications, referring MHCUs from the clinic to the district hospital for further management, and issuing referrals to social welfare for social grants. Additionally, psychiatric nurses arrange awareness campaigns on generic health related topics, and mental health is included in these as a sub-topic. All services are structured to address the needs of the MHCUs, with less focus on caregivers. The following comments give a clearer picture of the current situation:

“Most of them are the psychiatric trained nurses; those who have a qualification in psychiatry. We do not have the psychiatrists in our clinics; it is only the psychiatric nurses.” (MHC, SSI)

“Awareness campaigns are more comprehensive. It is where everything is included, HIV/AIDS, mental health, TB.” (PHCS, FG)

Some primary health clinics have CHWs to help extend services within the local community. CHWs work in collaboration with ward-based outreach teams and home-based carers to identify MHCUs within the community, monitor treatment compliance, and issue referrals to local clinics in cases where MHCUs are non-compliant. These services are focused on the MHCUs, with emphasis on monitoring treatment compliance. One participant explained, “Community health workers with the community ward-based outreach team are able to identify the mental healthcare users in the community, and they also monitor if they are complying with the treatment or not, and they refer them to the facility if they are not” (PHCS, FG).

School health services were identified as additional services within the community. These services are comprehensive and include mental health services. Nurses often conduct visits to schools within designated areas and render mental health services, including identification of learners who reside in the same house as MHCUs to offer them support and refer them where necessary. A participant said, “Some of the learners you find that there is a family member who is mentally ill, and it affects them because the community or other learners mock them at school. The nurses are able to identify those learners as a way of trying to find out what could be the problem” (PHCS, FG).

Service providers identified informal mental health services within the community and highlighted a lack of collaboration between informal and formal mental health service providers. Caregivers were reported to use informal services from traditional healers, churches, prophets, and pastors; for example, “We have the traditional healers and the people with mental health disorders are taken to them. We also have churches, prophets, pastors and the sangomas [traditional healers]” (PHCS, FG).

The service providers identified stakeholders within the community who support MHCUs and their caregivers. The stakeholders include the South African Police Services, who help caregivers take aggressive MHCUs to the hospital for treatment, and the South African Social Security Agency, who helps with the MHCUs’ disability grant. The following comments give more information on the stakeholders:

“The SAPS [South African Police Services] they assist us with the aggressive patients. If they are aggressive, they are the ones that take the user to the nearby health facility.” (MHC, SSI)

“We also refer to the SASSA [South African Social Security Agency] department because the patients should also apply for the grant.” (PHCS, FG)

Community leaders, including ward councillors and civic members, were also identified as stakeholders who help families get MHCUs to the hospital when they struggle, as the following comment explains:

“The community leaders like the civic members and the councillors are assisting. Like now we have a patient who has been brought by the councillor. Some of the community councillors are assisting in bringing the patients to us and to show support to the family, but this happens after the family has asked for assistance from them.” (MHP, SSI)

*Institution-based mental health services*. The service providers identified three referral hospitals offering mental health services within the Bushbuckridge community. Of the three hospitals, two offer 72-hour observation services and one offers in- and outpatient mental health services but only short-term care. Caregiver services include health talks, which are facilitated weekly during the outpatient clinic and focus mainly on improving the caregivers’ knowledge of mental conditions, the importance of treatment and compliance, monitoring the warning signs of relapses, and the importance of engaging MHCUs in daily occupations. The following comments expand on these services:

“The other facilities, which are [name of hospital] and [name of hospital], are offering 72-hour assessments, and that’s it. At least [name of hospital] is designated to keep them but not for a longest time; they keep them for a short time.” (MHP, SSI)

“We have a health talk with the caregiver and the patients when they come, and that generally focuses on the importance of taking their medication, warning signs of relapse, and also the importance of having a balanced daily occupational life at home.” (MHP, SSI)

Individual caregiver services are only offered on a referral basis. Referrals to a social worker, counsellor, or occupational therapists are issued if a caregiver expresses having difficulty caring for the MHCU; for example, “if the doctor sees that caregiver is really struggling, she would refer to us, and we would refer the caregiver to a social worker, OT [occupational therapist], or psychology” (MHP, SSI).

Interventions, such as a family therapy, that focuses on educating caregivers on mental disorders and the importance of treatment compliance are offered only when MHCUs are discharged from the hospital, and often occur with the person who fetches the MHCU from the hospital. One participant explained, “Family therapy happens only when the patient is admitted in the hospital, and they are getting the psychological service on referral when they are getting discharged. Not all family members are involved. The service is only given to the person who is there to fetch the patient home” (MHP, SSI).

#### Theme 3: Factors impacting delivery of caregiver mental health services

The service providers acknowledged that the current mental health services are inadequate to address the needs of informal caregivers. Various factors were identified as influencing the provision of caregiver-orientated mental health services, including personal, health system, and contextual factors.

*Caregiver-related factors impacting the delivery of caregiver mental health services*. The service providers indicated that informal caregivers are not always available. Despite their willingness to collaborate with informal caregivers in caring for MHCUs, service providers struggle as MHCUs often come for follow-up appointments on their own. One participant explained, “It is more important for us to collaborate with the caregivers, but we have found that sometimes the mental healthcare users come to the appointment alone and caregiver does not come with them. That also has been a challenge” (MHP, SSI).

Some informal caregivers reject help as they often hide the MHCUs from the CHWs when they conduct home visits to offer support. The participants indicated that CHWs are residents of the same community they serve and given the stigma informal caregivers experience within their communities, it can be difficult to receive help from members of the same community. One participant said, “The community health workers are not accepted in some of the families. If the community health workers visit them in order to give them support, the families hide their patients” (PHCS, FG).

Caregivers’ cultural beliefs were identified as another factor influencing the provision of caregiver services. Some informal caregivers seek help from traditional healers and religious organisations, which results in relapses among MHCUs as they stop taking their treatment, making their management challenging. One participant explained this challenge as follows:

“You want to help the client, but the family has other beliefs. They do not bring the client back, and if they come back, they present with severe psychotic features, and this could be prevented if the person adhered to medication. So, culture is the challenge, and we have to look at how best do we bridge this gap and for us to be able to bridge it. We are also a great team.” (MHC, SSI)

*Health system factors impacting the delivery mental health services to the caregiver*. The service providers acknowledged that services focus on addressing physical conditions rather than mental disorders. Support groups are regularly facilitated for those with HIV, TB, and diabetes, but none of the support groups focus on those with mental disorders. A participant admitted this, saying, “I can see that the mental health issue is being overlooked, and I am saying this because we do have the diabetic support groups, HIV and TB support groups, and also the disability support group” (PHCS, FG). As a result, caregivers of MHCUs only receive support upon request, which is concerning considering that most informal caregivers lack knowledge and often delay seeking help for the MHCUs. One participant explained the situation by saying, “The support is only given if the caregiver goes to the clinic and report a case that she needs help with, and the home-based outreach attends to that case or the OTTs [Occupational Therapy Technicians]. This happens only if the caregiver comes to ask for assistance. We don’t have people going out to ask caregivers if they are coping” (PHCS, FG). The stigma and limited knowledge among some of the lay workers, including CHWs and home-based carers, cause some to be afraid of MHCUs, and therefore, reluctant to assist caregivers as they perceive the MHCUs to be violent and dangerous. One participant explained that “the community-based workers are usually afraid of these patients. It is the stigma. They feel that these people are very dangerous” (MHP, SSI).

The service providers also indicated a lack of referral pathways between the hospital and community-based services. Some of the service providers are not always aware of the lay workers in the community, and there are no established referral pathways to enable them to refer both the MHCUs and their caregivers. This breakdown in communication influences the continuity of care from hospital to the community. A participant said, “We do not have a system of referring directly, and that would be useful because we are good at giving referrals to the clinics, and we might use the home-based carers, but we do not know where the active home-based carers are and that information would be useful as well” (MHP, SSI).

The service providers indicated that budget constraints in mental health is an ongoing problem that affects the provision of mental health services. Limited access to physical resources, such as transport to conduct home visits, was identified as a factor that influences service providers’ ability to support informal caregivers. A participants explained it as follows: “We have transport problem, but if we had transport, we would be able to visit the families to check if the user is taking the treatment and to check the relationship between the user and the family” (MHC, SSI).

*Contextual factors impacting the delivery of caregiver mental health services*. While the service providers acknowledged the important role played by stakeholders such as the South African Police Services to help them support caregivers, they also indicated that lack of training among the police limits their ability to handle the MHCUs appropriately. One participant explained that “another thing as far as SAPS [South African Police Services] is concerned, you find that some of the police are not empowered on how to handle the mental healthcare user’s cases” (MHP, SSI).

The distance travelled to the hospital was identified as a problem. The fact that only one hospital has a mental health unit within Bushbuckridge means that some informal caregivers need to travel long distances to access the services they require. This poses a challenge as some informal caregiver’s experiences financial strain, and one participant said, “The hospital is the one that is challenging because they need money to travel to the hospital” (MHP, SSI).

The rurality of Bushbuckridge municipality makes it difficult to attract skilled mental health professionals like psychologists, psychiatrists, and social workers, and this reduces access to these services within the community. One participant explained the challenge as follows: “Our challenge is that we have posts that are funded for clinical psychologists, but we don’t have people who can come and fill them. This is because these areas are rural, and we have young people that are coming from the universities. They wouldn’t opt to go to such places” (MHC, SSI).

## Discussion

The study aimed to explore the perspectives of service providers regarding the experiences of informal caregivers of persons with SMDs and the mental health services available for supporting these caregivers in their role. Three themes were identified, namely perceived caregiving consequences and related factors, current state of mental health services, and factors affecting delivery of mental health services to the caregiver. Understanding the service providers’ perspectives on the challenges that are encountered by the informal caregivers in their caregiving was useful for establishing their awareness and their understanding of the need for caregiver-orientated mental health services. Additionally, this helped establish a more comprehensive view of the burden of care among informal caregivers, which is vital for planning and developing contextually responsive strategies for alleviating informal caregiver burden.

### Factors impacting on caregiving

The service providers in this study indicated that informal caregivers of persons with SMDs encountered challenges in their caregiving role which led to stress and burden. These challenges were attributed to the MHCUs functional limitations, aggressive and violent behaviours, and substance abuse. Due to their functional limitations, some MHCUs require full-time care, and as a result, informal caregivers had to leave their jobs, which led to financial strain that predisposes informal caregivers to economic burdens. These findings are consistent with previous studies conducted on informal caregiver experiences in sub-Saharan Africa that reveal that the caregivers’ employment status and the severity of the MHCUs’ condition are associated with the economic burden experienced by the caregivers [26, 41]. The fact that some caregivers are minors who had to leave school to look after their parents with a SMD raises a serious concern regarding the well-being and future of these minor caregivers. Leahy [42] highlights that children whose parents are diagnosed with SMDs must undertake inappropriate levels of responsibility as they are often expected to care for themselves, their siblings, as well as look after the household in addition to caring for their mentally ill parents. Consequently, these children are at risk of developing psychological, social, and academic problems [42]. Leaving school early reduces the chances of entering the open labour market, particularly in resource-constrained areas like rural South Africa where fewer economic opportunities exist [43]. It can therefore be concluded that occupying this role plunges these minor caregivers further into the cycle of poverty [18].

The service providers highlighted substance abuse as a common problem among the MHCUs, which is related to high relapse rates that subsequently lead to aggressive and violent behaviours, making it difficult for informal caregivers to provide care. The findings are similar to that of previous studies that reveal that high relapse rates among persons with SMDs are mainly related to substance abuse and that this contributes largely to the high levels of caregiver burden [29, 44].

In agreement with the concerns raised by informal caregivers in previous studies conducted in rural South Africa and Zimbabwe, the service providers identified stigma as a contributing factor to caregiver burden [26, 45]. Similar to previous studies conducted in rural South Africa, the service providers expressed that caregivers are susceptible to exploitation by community members who expect them to cover costs for damages caused by MHCUs during their psychotic states [30]. The experience of stigma exposes caregivers to feelings of loneliness and isolation, subsequently leading to negative consequences for the caregivers’ emotional, mental, and physical health. Some informal caregivers were reported to have consulted mental health professionals for depression, anxiety, and adjustment disorders. In addition, others reported having developed physical conditions, such as high blood pressure, which is consistent with the findings highlighted by informal caregivers in previous studies [45–47].

Various factors are related to the burdens and negative consequences experienced by informal caregivers in their role. Caregiver-related factors, including caregivers’ lack of knowledge and skills in handling MHCUs, were attributed to burdens expressed in this study. These results are supported by Ntsayagae et al. [48] and Marimbe et al. [45], who reveal that informal caregivers reported lack of knowledge on mental conditions and practical skills required to assist MHCUs, and as a result, they struggle to cope as they feel powerless in their caregiving roles. Similarly, the service providers in this study reported that informal caregivers lack information on mental conditions, which compromises their ability to cope as they struggle to handle the behaviours exhibited by the MHCUs. The service providers perceived that lack of knowledge of mental disorders leads informal caregivers to delay help-seeking as they often wait until the MHCUs are aggressive and violent to seek help. Lack of knowledge on mental disorders is considered a common problem that influences help-seeking behaviour in LMICs [49, 50]. This is because the approach to decentralise mental health services was adopted without the proper development of community-based mental health services, and as such, there is limited awareness of mental disorders that prevail within communities [51]. A study conducted in rural South Africa reveals that lack of knowledge about mental illness is a factor that influences help-seeking behaviour among MHCUs and their caregivers, and recommends psychoeducation for caregivers as a way of addressing this problem [52]. Chadda [22] highlights that in order for psychoeducation to be effective, sessions must be held weekly for a period of three months to one year and should include basic information on a mental disorders, common symptoms, available treatments, side effects, and warning signs of relapses. In addition, these interventions should include practical strategies for the day-to-day handling of patients, as well as stress management and coping skills for the caregivers [22].

Contextual and intersectoral factors such as limited access to community resources, including long-term facilities and day centres to rehabilitate MHCUs, were highlighted as contributing factors to the burdens and consequences encountered by informal caregivers. Service providers expressed that MHCUs receive hospital treatment that stabilises their conditions but that there is no continuity of treatment to rehabilitate them within their communities. According to Saha et al. [53], rehabilitation of MHCUs is critical for facilitating the development of the functional, social, and intellectual skills they require to lead independent lives within their communities. Additionally, the World Health Organization commends community-based psychiatric rehabilitation as a core element in improving quality of life and facilitating inclusion and participation among MHCUs within communities [54]. The limited access to rehabilitation services compromises MHCUs’ ability to gain the necessary skills for independent living. It is therefore not surprising that in this study informal caregivers were reported to provide full-time care to their care recipients with SMDs, which predisposes them to economic burdens.

### Current state of mental health services

Despite acknowledging the burdens faced by informal caregivers and the negative consequences of caregiving on the emotional, mental, and physical health of caregivers, the current mental health services do not address the needs of caregivers. While service providers identified some institution-based caregiver services, such as health talks, psychoeducation, and family therapy, these services were reported to be made available on interim basis and mainly directed at improving the MHCUs well-being with less focus on caregiver health and wellness. Additionally, these services are targeted specifically at caregivers who already presented with a problem, highlighting that the services follow the biomedical approach and place less emphasis on prevention and health promotion among informal caregivers. Referrals to mental health professionals, including social workers, counsellors, and occupational therapists, are only done in cases where caregivers express difficulty coping with their duties. This raises serious concerns because informal caregivers often lack information about the services available for them. These findings are consistent with those reported by Akbari et al. [55], who reveal that the health status of caregivers is often not prioritised in mental healthcare. The lack of prioritisation of caregivers’ health means that their unique needs are not considered, which perpetuates their burdens and health risks [55].

The service providers identified non-specialist health workers, including CHWs and home-based carers, as key role players in the management of mental disorders within the community. A study conducted in five LMICs, namely Ethiopia, India, Nepal, South Africa, and Uganda, reveal that using non-specialist health workers is an acceptable and feasible way to improve access to mental health services in low-resourced communities [56]. The availability of non-specialist health workers within the study context is an opportunity to extend community mental health services to help alleviate burdens among informal caregivers of persons with SMDs.

The service providers in this study identified informal mental health service providers within the community, including traditional healers, herbalists, and religious organisations, but highlighted a lack of collaboration between these service providers and the formal mental health service providers within the community. Sorsdahl et al. [57] identify alternative practitioners, such as traditional healers, as key role players in the delivery of mental health services. This emphasises the need for collaboration between mental health workers and alternative service providers such as traditional healers and religious practitioners, particularly in low-resourced settings where informal caregivers have been reported to rely on their religious practices to help cope with the demands of caregiving [10, 58, 59].

Community-based stakeholders, including the police and community leaders, including ward councillors and civic members, were identified as a valuable resource that help families of persons with mental disorders during mental health emergencies. These stakeholders were identified to be helpful in assisting families to get aggressive MHCUs to the hospital when needed. These findings are consistent with those reported by Modise et al. [59] in rural South Africa where families of MHCUs identified police intervention as a critical resource that help them cope in emergency situations where MHCUs are uncontrollable.

### Factors affecting the delivery of caregiver mental health services

Service providers acknowledged that the current institution-based and community mental health services are inadequate to address the burdens experienced by informal caregivers of persons with SMDs. The unavailability of caregivers, rejection of help from the CHWs, and caregivers’ cultural beliefs were identified as caregiver-related factors influencing the delivery of caregiver mental health services. Some of the institution-based service providers expressed their interest in working collaboratively with informal caregivers to support them in their caregiving role; however, they encounter a challenge as informal caregivers are often unavailable during follow-up visits. The unavailability of informal caregivers could be attributed to the transport costs incurred in accessing institution-based mental health services. In addition, this could be because informal caregivers occupy other roles and have responsibilities outside of their caregiving duties, which make it difficult for them to attend follow-up visits with the MHCUs. The fact that only one hospital has a mental health unit that offers in- and outpatient services means that some need to travel long distances to access services for themselves and the MHCUs. Previous studies conducted in rural South Africa reveal high levels of financial burdens [26–29]. Given that the services are tailored to meet the needs of the MHCUs with less focus on the caregivers’ health and well-being, it can be assumed that once the informal caregivers perceive the MHCUs to be stable enough to attend follow-up visits, they stop accompanying them to cut the travelling costs. Ntsayagae et al. [48] reveal that informal caregivers are required to combine their caregiving duties with other roles and responsibilities, such as executing daily chores and taking care of themselves and other family members. To ensure successful collaboration with informal caregivers in the care of MHCUs, service providers should direct services towards assisting informal caregivers to obtain balance in their activities. The rejection of help from CHWs could be related to the stigma that informal caregivers encounter within the community. The CHWs usually render services to the same community in which they reside [60], and therefore, it could be that informal caregivers are reluctant to receive help from members of the same community that blame them for the behaviours exhibited by the MHCUs. It is therefore important to ensure mental health education to orientate informal caregivers to mental health services that are available and their purpose in alleviating their burdens. In addition, stigma reduction programs are required to address the exploitation of informal caregivers by the community [50].

The cultural beliefs of the informal caregivers were identified as a caregiver-related factor that hinders delivery of support to informal caregivers. Service providers indicated that some informal caregivers prefer services from traditional healers and religious organisations, which impedes caregiver support and MHCUs’ recovery. According to Cauce et al. [12], cultural beliefs have a substantial influence on help-seeking and choice of treatment providers. Even though the informal caregivers in this study seek medical help, they still consult traditional healers, who stop MHCUs’ from taking medication, which worsens their condition. This highlights the importance of adopting a collaborative care model where mental health professional and complementary service providers like traditional healers ensure the recovery of MHCUs and build support for informal caregivers [10, 49, 61].

Health system factors, including limited prioritisation of mental health services, limited knowledge and stigma from lay health workers, lack of referral pathways between institution-based and community-based mental health service providers, and funding constraints in mental health, were identified as influencing the provision of caregiver-orientated mental health services. The service providers acknowledged that mental health, particularly that of caregivers, is not prioritised within the community. In addition, they highlighted continuous budget constraints from the Department of Health in South Africa, which hinder outreach mental health services in the community. Matlala et al. [62] state that while mental health is considered a critical component of health, there is a general neglect of mental health services evident in LMICs. It is for this reason that even with the high prevalence of mental disorders, these countries are reported to spend less than 3% of their budget on mental healthcare [63]. It is therefore not surprising that budget constraints were highlighted as influencing the provision of caregiver services in this study. Rathod et al. [10] highlight the need for prioritising mental health services and emphasise that this can only be achieved with political will, improved legislation, equitable resource distribution, and collaborations between MHCUs, caregivers, and formal and informal health providers. While service providers highlighted caregivers’ rejection, of services from CHWs, they also highlighted that lay workers, including CHWs and home-based carers, often avoid providing services because of the stigma that MHCUs are violent. According to Sibeko et al. [9], CHWs received basic training on chronic conditions such as HIV, hypertension, and diabetes but no standard training on mental health. It can therefore be concluded that the attitudes displayed by lay health workers in this study context can be attributed to the limited knowledge related to lack of training on mental health. Contextual factors, such as limited knowledge from stakeholders such as the South African Police Service, highlight the need for training to ensure better handling of MHCUs and support to caregivers [59]. Distances travelled to hospital and rurality of the study context were reported as contextual factors influencing the delivery of caregiver interventions. Service providers indicated that the provision of institution-based mental health services pose a challenge since informal caregivers must travel long distances and struggle because of financial constraints. The fact that the study context is in a rural area makes it difficult to attract skilled mental health practitioners [64]. The contextual factors highlight the importance of strengthening community-based mental health services to ensure access of services to informal caregivers. The gap in mental health professionals can be bridged by integrating mental health into the training of CHWs and home-based carers [8, 65].

### Limitations

The study only focused on investigating caregiver mental health services within the Bushbuckridge municipality, and while there may be similarities of the study context to allow for replication of findings in other contexts, the findings cannot be generalised to all rural areas of South Africa. While conducting interviews in the participants’ workplace was useful for understanding their work context, it also meant many interruptions during the interviews, which could have influenced the depth of information shared by the participants. In cases where there were interruptions, the interviewer requested an additional time for the interview to allow the participants enough time to share their views. Member checking was also conducted to ensure that participants had an opportunity to review their transcripts and add any relevant information that the participants wished to share. In one of the interviews, it was mentioned that minor children are caregivers; however, this was reported by the service providers who could not trace the information back to where these children are to provide proper support. The lack of convergent systemic and culturally competent evidence evincing the likelihood ratios of SMDs might have contributed towards potential interviewer and responder bias.

## Conclusions

The results revealed that service providers acknowledged the challenges encountered by informal caregivers of persons with SMDs in their caregiving role. The challenges encountered by informal caregivers result in negative consequences that influence caregivers’ emotional, mental, and physical well-being. Despite the service providers’ awareness of the challenges encountered by informal caregivers and the subsequent burdens, they struggle to provide caregiver-orientated mental health services because of personal, health system, and contextual factors. Given the continuous challenges facing the provision of mental health services in low-resourced contexts, it is necessary to explore strategies to facilitate the efficient use of existing resources. The findings in this study calls for the development and strengthening of community-based rehabilitation of MHCUs to improve their function within the community and reduce their dependency on their informal caregivers. Strategies such as task shifting can be adopted to train lay workers, including CHWs and home-based carers, to extend rehabilitation services within the community. Training lay workers should also include knowledge and skills on the delivery of caregiver-orientated services to improve support for informal caregivers. Informal caregivers should be educated on available services to improve their help-seeking and acceptance of help from lay workers in the community. Future studies should focus on investigating collaborations between formal and informal mental health service providers and stakeholders within the community to ensure the provision of user-friendly and accessible mental health services for informal caregivers.
